# Common Mental Disorders and Economic Uncertainty: Evidence from the COVID-19 Pandemic in the U.S.

**DOI:** 10.1371/journal.pone.0260726

**Published:** 2021-12-02

**Authors:** Wing Wah Tham, Elvira Sojli, Richard Bryant, Michael McAleer

**Affiliations:** 1 UNSW Business School, University of New South Wales, Sydney, New South Wales, Australia; 2 School of Psychology, University of New South Wales, Sydney, New South Wales, Australia; 3 College of Management, Asia University, Taichung, Taiwan; University of Jyvaskyla, FINLAND

## Abstract

Mental health disorders represent an enormous cost to society, are related to economic outcomes, and have increased markedly since the COVID-19 outbreak. Economic activity contracted dramatically on a global scale in 2020, representing the worst crisis since the Great Depression. This study used the COVID Impact Survey to provide insights on the interactions of mental illness and economic uncertainty during COVID-19. We used a probability-based panel survey, COVID Impact Survey, conducted in the U.S. over three waves in the period April-June 2020. The survey covered individual information on employment, economic and financial uncertainty, mental and physical health, as well as other demographic information. The prevalence of moderate mental distress was measured using a Psychological Distress Scale, a 5-item scale that is scored on a 4-point scale (total range: 0–15). The mental distress effect of employment, economic, and financial uncertainty, was assessed in a logit regression analysis conditioning for demographic and health information. It is found that employment, health coverage, social security, and food provision uncertainty are additional stressors for mental health. These economic factors work in addition to demographic effects, where groups who display increased risk for psychological distress include: women, Hispanics, and those in poor physical health. A decrease in employment and increases in economic uncertainty are associated with a doubling of common mental disorders. The population-representative survey evidence presented strongly suggests that economic policies which support employment (e.g., job keeping, job search support, stimulus spending) provide not only economic security but also constitute a major health intervention. Moving forward, the economic uncertainty effect ought to be reflected in community level intervention and prevention efforts, which should include strengthening economic support to reduce financial and economic strain.

## Introduction

Mental health disorders represent an enormous cost to society because of healthcare outcomes, insurance payments, lost productivity, and unemployment. The World Economic Forum has projected that by 2030 mental health disorders will account for more than half of the global economic burden due to non-communicable diseases, estimated at approximately US$6 trillion annually [1]. Given their greater prevalence, common mental disorders (CMDs) such as depression, anxiety, and substance abuse represent a major burden on society. One Australian study [2] found that the estimated annual cost of common mental disorders was approximately AUD$12 billion dollars. Another national study [3] found that, whereas depression carries the major economic cost, a greater prevalence of anxiety results in CMDs accounting for a larger absolute economic burden.

Recent meta-analyses and systematic reviews demonstrate that approximately one in three people have experienced psychological distress during the pandemic (e.g., [4, 5]). Cohort studies indicate that the rates of common mental disorders have increased during COVID-19 pandemic, with the greatest increases in those with no prior history of psychological problems. Several COVID-19 related studies show that the physical distancing and stay-at-home orders have led to elevated loneliness in many people [6] and higher levels of CMDs especially in young adults [7].

The pandemic has also severely and adversely affected the demand and supply sides of the economy, and economic activity contracted dramatically on a global scale in 2020, representing the worst crisis since the Great Depression, the worst economic crisis in recent world history [8]. The World Economic Outlook July report showed a decrease of 3.2% in economic growth for 2020 and very uncertain economic recovery for 2021 [9]. The Poverty and Shared Prosperity [8] projects an increase in extreme poverty by 1.4% (88 million people) and increased economic uncertainty for those in poverty (below $5.5 a day) in 2020. 55% of the world population lives in poverty, the overwhelming majority of which is in developing countries, but 20% lives in developed countries [8]. Previous work shows that a strong relation exists between mental health care needs and provision and economic crises (see survey in [10]). The World Health Organization (WHO) has already documented that 60% of 130 countries have experienced disruptions in mental health services during the pandemic [11].

In this context, it is worth noting the strong evidence that economic difficulties are typically related to worse mental health outcomes. Suicide rates increase during periods of economic downturn [12], with one report demonstrating that each 1% increase in unemployment is associated with a 1% increase in suicide rates [13]. The association between poverty and mental illness is complex [14], and economic disadvantage is associated with a greater likelihood of mental illness [15]. The economically disadvantaged are often vulnerable to the downward spiral of increased stressors associated with poverty and unemployment [15, 16]. Furthermore, mental disorders with an early onset affect the success in the labor market over the whole life course [17].

These findings highlight the need to identify the most vulnerable groups to psychological illness, and the determinants of their distress, to administer effective public healthcare policy. Unfortunately, we cannot employ similar public policies from previous epidemics or pandemics, such as Severe Acute Respiratory Syndrome (SARS) and Middle East Respiratory Syndrome (MERS), as the healthcare and economic impacts of COVID-19 are well beyond what has been experienced in over a century. COVID-19 has infected, and continues to infect, the world population at record speed, with increasing rates of spread, infection, and mortality and has created a unique mix of public health and economic challenges. [18] called for multidisciplinary research to develop mental-health responses during the pandemic, while [15] emphasized the need for further evidence on the intertwined relation between economics and mental health. In summary, it is critical to identify the fundamental drivers of mental distress during the COVID-19 pandemic to implement safe and effective public healthcare policies and provide occupational guidelines to the population, especially to the more vulnerable groups.

The primary purpose of the current study is to use the COVID Impact Survey conducted by the National Opinion Research Center (NORC) at the University of Chicago to provide insights on the interactions of mental illness and economic uncertainty during COVID-19. The data are different from most mental health surveys that have been undertaken during COVID-19, because they are based on a national probability-based sample that permits conclusions to be drawn about the relation among COVID-19, economic effects, and mental health. The survey is dedicated to understanding the impact of COVID-19 and related measures, which include several questions on individual economic conditions and uncertainty.

Although there has been a plethora of studies reporting an increase in the incidence rates of CMDs during COVID-19 [19, 20], and work documenting the relation between economic outcomes and mental health in other settings, this study supplements this evidence by integrating mental health responses with economic uncertainty data. Prior literature has not investigated the role of economic uncertainty in mental health outcomes. Our two main research questions were: Is there a relation between economic uncertainty and mental health outcomes? Which types of economic uncertainty matter for mental health outcomes and what is the magnitude of the relation?

## Methods

### Survey details

The COVID-19 Household Impact Survey is a philanthropic effort to provide national and regional statistics about physical health, mental health, economic security, and social dynamics in the U.S. during COVID-19, as described in detail in [21–23]. The COVID Impact Survey is funded by the Data Foundation and conducted by the NORC at the University of Chicago. The survey targets a nationally representative sample of U.S. adults aged 18 and older. The sample is selected from the panel using sampling strata. Data comes from a probability-based panel designed to be representative of the U.S. household population, which is in stark contrast to any other mental health survey conducted during the pandemic. The size of the selected sample per sampling stratum is determined by the population distribution for each stratum. In addition, the sample selection accounts for expected differential survey completion rates by demographic groups, so that the set of panel members with a completed interview for a study is a representative sample of the target population. For technical information about the data, including the recruitment process and panel management policies, see the Appendices in [21–23].

The analysis used the three available waves of the survey carried out on: 20–26 April, 4–10 May, and 30 May-8 June 2020. The samples are drawn independently for each week to provide independent, cross-sectional estimates for each region and wave. Participants provided informed consent when they joined the NORC panel and were informed that their identities would remain confidential. For phone respondents, the statement was read aloud. For online respondents, the statement was presented on the screen. All research activities and consent procedures were reviewed and approved by the NORC Institutional Review Board for Human Subjects research.

### Mental wellness

The five mental health questions we analyze are: In the past 7 days, how often have you: 1) felt nervous, anxious, or on edge; 2) felt depressed; 3) felt lonely; 4) felt hopeless about the future; 5) had physical reactions such as sweating, trouble breathing, nausea or a pounding heart when thinking about your experience with the coronavirus pandemic?

The response options were: (1) Not at all or less than 1 day, (2) 1–2 days, (3) 3–4 days, or (4) 5–7 days. For each question, a value of zero, one, two, or three was assigned to each answer, respectively. The mean score for questions (1)–(4) is 0.64 (s.e. = 0.01), and 0.15 (s.e. = 0.01) for question 5, see panel A of S1 Table in S1 File. Responses to the five symptoms were aggregated to yield a composite score with a range from zero to 15, where higher scores indicate a greater tendency towards CMDs. We refer to the composite score as T5. The distribution of the T5 composite score is presented in S1 Fig in S1 File.

### Validity check

The mental health constructed variables are highly correlated with each-other, with correlations varying between 0.36 (p-val. < 0.01) and 0.57 (p-val. < 0.01). They are also highly correlated with the composite score T5, with correlations varying between 0.57 (p-val. < 0.01) and 0.81 (p-val. < 0.01), in panel B of S1 Table in S1 File. The Cronbach alpha results demonstrate excellent internal consistency and reliability, as the raw and standardized alpha scores are around 0.82 in panel C of S1 Table in S1 File. All the variables are equally important for the internal consistency of a composite measure, in panel D of S1 Table in S1 File.

The computed T5 composite score was compared against a criterion measure based on previous mental health diagnosis. We use question PHYS8H:

‘Has a doctor or other health care provider ever told you, you have any a mental health condition?’

Respondents that answered ‘YES’ were categorized as needing mental health treatment, and this is our criterion measure.

The receiver operating characteristic (ROC) curve analysis was used to validate the T5 composite score against the criterion measure. The ROC curve is a plot of the true positives (sensitivity) versus the false positives (1 –specificity) for a binary clinical outcome classifier system, as its screening test discrimination threshold is varied. We calculated the area under each ROC curve (AUC), which ranges from 0.5 to 1, with 1 indicating perfect discrimination. The AUC area can be interpreted as the probability that randomly chosen respondents, with or without the clinical outcome of interest, would be distinguished correctly based on their screening test scores [24]. The clinical outcome in this validation indicates whether the respondent reported that a doctor or other health care providers ever told them that they have a mental health condition, and the screening test is the T5 scale.

It is observed that 15.21% of respondents reported that a doctor or other health care providers ever told them that they have a mental health condition. These individuals have a T5 score with mean = 5.61 (s.e. = 0.12), compared with those who did not report that a doctor or health care provider has told them that they have a mental health condition, with mean = 2.08 (s.e. = 0.039). The full sample mean for the T5 score is 2.65 (s.e. = 0.042).

We identify the optimal T5 threshold for classification of moderate mental distress using the ROC curve analysis for the full sample, see S2 Fig in S1 File. We use the cut-off point that maximizes Youden’s J statistic (sensitivity + specificity -1). The optimum threshold is T5 ≥ 3, where sensitivity = 0.70, specificity = 0.73, total classification rate = 0.70, and AUC = 0.77, which indicate a high level of accuracy. Thus, we created a moderate mental distress indicator equal to one if T5 ≥ 3, and zero otherwise. Additional analysis was carried out using the continuous scale variable (T5) and different cut-off points for the moderate distress indicator (score ≥ 4, score ≥ 5). Statistical inference and conclusions did not change in the additional analysis.

### Explanatory variables

Apart from the demographic (gender and race), household composition (alone, with other adult, with 1–2, or more children) and income information provided by the survey respondents, respondents were also asked about their employment, economic, and financial circumstances through the following questions.

#### Employment

“**In the past 7 days, did you do any work for pay at a job or business?**” with response options: “Yes, I worked for someone else for wages, salary, …,” “Yes, I worked as self-employed in my own business, …,” “No, I did not work for pay last week.” The latter was used as the benchmark. This question was followed by “**How many hours did you work last week at all jobs?**” and “**Prior to March 1, 2020 when COVID-19 began spreading in the United States, how many hours did you usually work each week?**” where respondents inputted the number of hours worked. To capture an individual’s change in working hours due to the pandemic, we constructed a continuous variable. The change in working hours is the difference in the hours worked in the week prior to the survey (that is, during the pandemic) and the hours worked in a typical week prior to March 1, 2020 (that is, mean working hours prior to the pandemic).

“**Think about 3 months from now, how likely do you think it is that you will be employed at that time?”** with response options: “Extremely likely,” “Very likely,” “Moderately likely,” “Not too likely,” “Not likely at all.” The latter was used as the benchmark.

#### Economic security

“**In the past 7 days, have you either received, applied for, or tried to apply for any of the following forms of income or assistance, or not?**” with response options: “Received,” “Applied for,” “Tried to apply for,” “Did not receive nor apply for any.” The latter was used as the benchmark. The questions were asked in reference to: Social security, Unemployment insurance, and Any kind of government health insurance or health coverage plan including Medicaid, Medical Assistance or Medicare using the exact same wording.

#### Financial security

“**[We worried our food would run out before we got money to buy more] Please indicate whether the following statements were often true, sometimes true, or never true for you or your household over the past 30 days.**” with response options: “Often true,” “Sometimes,” “Not at all.” The latter was used as the benchmark.

#### Other variables

In addition, respondents were asked about their communication with others and the state of their physical health through the following questions: “**In the past month, how often did you communicate with friends and family by phone, text, email, app, or using the Internet?**” “**During a typical month prior to March 1, 2020, when COVID-19 began spreading in the United States, how often did you communicate with friends and family by phone, text, email, app, or using the Internet?**” (response options for both: “Basically every day,” “A few times a week,” “A few times a month,” “Once a month,” “Not at all”. The latter was used as the benchmark.) and **“Would you say your health in general is excellent, very good, good, fair, or poor?”** (poor physical health was used as the benchmark).

### Statistical analysis

We quantified the relation between economic uncertainty and CMDs through univariate comparisons and multivariate regressions, with the dependent variable being the moderate mental distress indicator. The analysis was carried out using SAS 9.4. First, we formally analyze the relation between the change in working hours and moderate mental distress. We estimate a multivariate logistic regression of the moderate mental distress variable on the change in working hours controlling for individual demographic characteristics (gender, race, age, household composition and income). We use a pooled regression, as the individual survey respondents are drawn independently. Second, we estimate a multivariate logistic regression of the moderate mental distress indicator and the economic and financing variables. The explanatory variables were the employment, economic uncertainty, and financial uncertainty variables. Potential confounding effects were controlled for through covariates for gender, race, age, household composition and income, prior mental health diagnoses, physical health, contact with others prior and during the pandemic. We also controlled for the survey wave period using survey wave fixed effects, where the first survey in April 2020 was the benchmark period. We use population sampling weights for the regression analysis.

## Results

The sample characteristics and moderate mental distress prevalence rates across various subgroups are discussed in Supplementary Material section S1 in S1 File.

### Univariate analyses

We focus on the relation among employment, economic, and financial needs of the survey participants and moderate mental distress. All other relations are presented in S3 Table in S1 File. The mean number of working hours in comparison to pre-COVID-19 has decreased by 3.5 hours a week, with standard deviation of 11 hours (S4 Table in S1 File).

**Fig 1:**
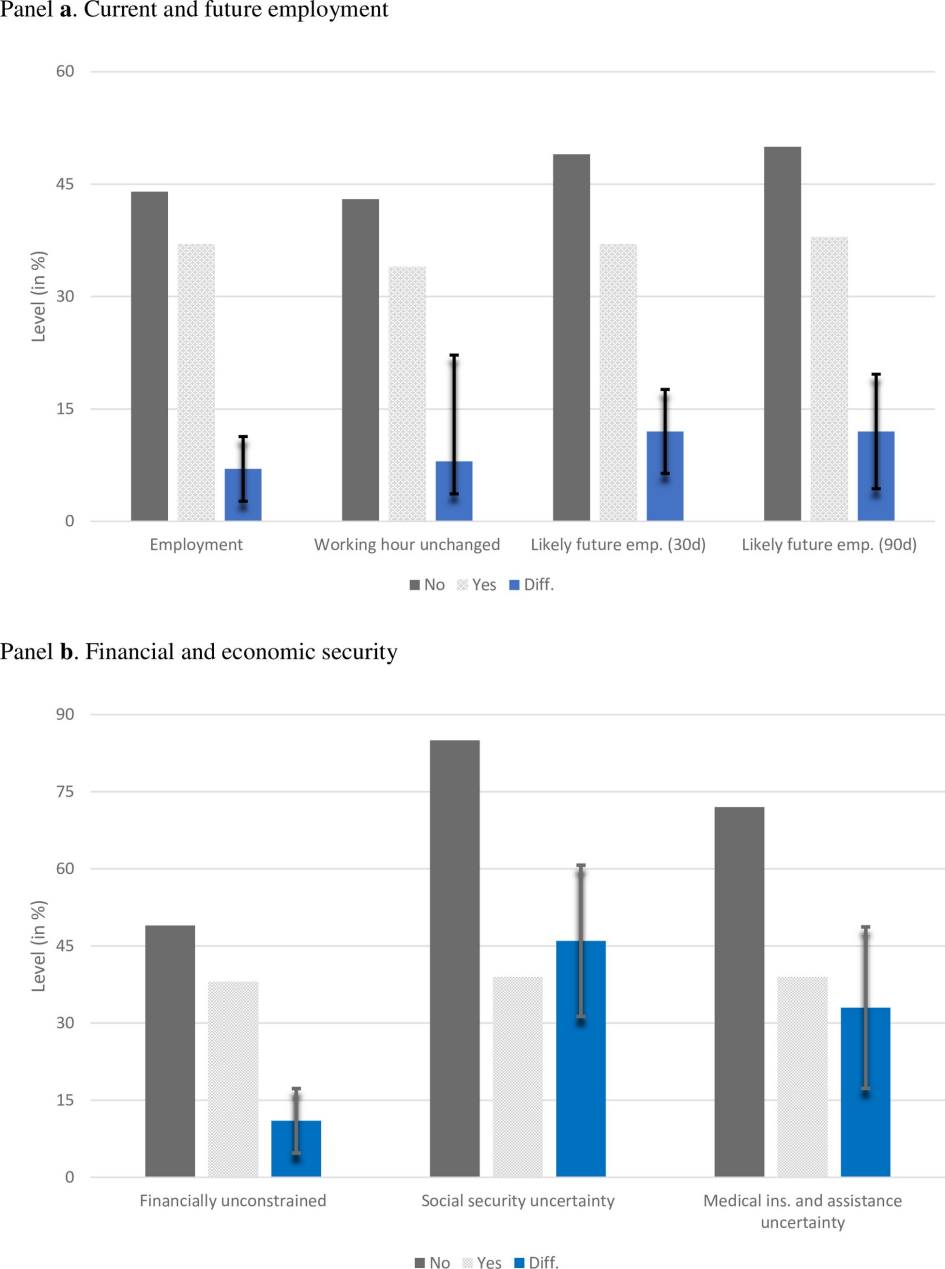
Moderate mental distress, employment, financial and economic security. Panel **a** presents the levels (%) and differences in moderate mental distress for current and future employment variables. *Employment* refers to responses to the question: During the past 7 days were you employed? *Working Hour Unchanged* is No when the difference between the hours worked in the prior week and prior to 1 March 2020 is less than zero. *Likely future emp*. *(30d/90d)* refers to responses to the question: Think about 30 (90) days from now, how likely do you think it is that you will be employed at that time? Responses in the categories: (4) not too likely and (5) not likely at all, are classified as No and the rest as Yes. Panel **b** presents the levels and differences in moderate mental distress for financial and economic security variables. *Financial unconstrained* refers to responses to the question: Based on your current financial situation, how would you pay for a $400 expense? Responses are categorized as Yes if respondents can pay cash, from bank account, or fully cover the credit card fee, and No otherwise. *Social security uncertainty* classifies individuals that have applied, received, or do not need social security as Yes and the others as No. *Medical ins*. *and assistance uncertainty* classifies individuals that have applied, received, or do not need social security as Yes and the rest as No. The grey bars present the levels, the blue bars present the differences between the Yes and No categories, and the error bars present the 95% confidence intervals.

Panel a of Fig 1 shows the differences in employment, working hours, and the likelihood of finding work in the next 30 and the next 90 days for respondents exhibiting moderate mental distress (where score T5 ≥ 3). Employment refers to responses as yes or no to the question: During the past 7 days were you employed? Working Hour Unchanged is codified as Yes when the difference between the hours worked in the prior week and prior to 1 March 2020 is zero and No when the difference is less than zero. Likely future emp. (30d/90d) refers to responses to the question: Think about 30 (90) days from now, how likely do you think it is that you will be employed at that time? Responses in the categories: (4) not too likely and (5) not likely at all, are classified as No and the rest as Yes. 44% of the unemployed reported moderate mental distress as compared to 37% of the employed respondents. Around 44% of respondents whose working hours were reduced during the pandemic also suffered from moderate mental distress compared to 34% of those whose working hours were not changed. There is a 50% prevalence rate of moderate distress for those whose likelihood of having employment 30 or 90 days following the survey is low (the no category) compared to 38% for the others. Differences between the Yes and No respondents, presented in blue boxes, ranged from 6–12% and were statistically different from zero, using t-tests. The 95% confidence intervals are presented as error bars.

Panel b of Fig 1 presents the differences in moderate mental distress for respondents with financial and economic security, and for those without. Respondents who needed/wanted to file for social security and/or medical insurance but did not manage to do so (the No category), exhibited the highest proportion of moderate mental distress. Specifically, these respondents were 40–45% more mentally distressed than those who did not apply, applied, and received help from the various safety nets. The differences remain when using a threshold of 4 or 5 for the moderate distress scale as presented in S3 Fig and S5 Table in S1 File.

### Multivariate analysis

The empirical analysis presented so far is univariate, but several of these variables may be mediated or confounded by interactive demographic and individual characteristics. The estimated results in S5 Table in S1 File show that the elasticity, or proportionate effect, is always significant, both statistically and economically (even conditioned on a large set of control variables). A decrease of one standard deviation (11 working hours) per week increased the probability of an individual feeling moderate distress by 1.54% per week.

**Fig 2:**
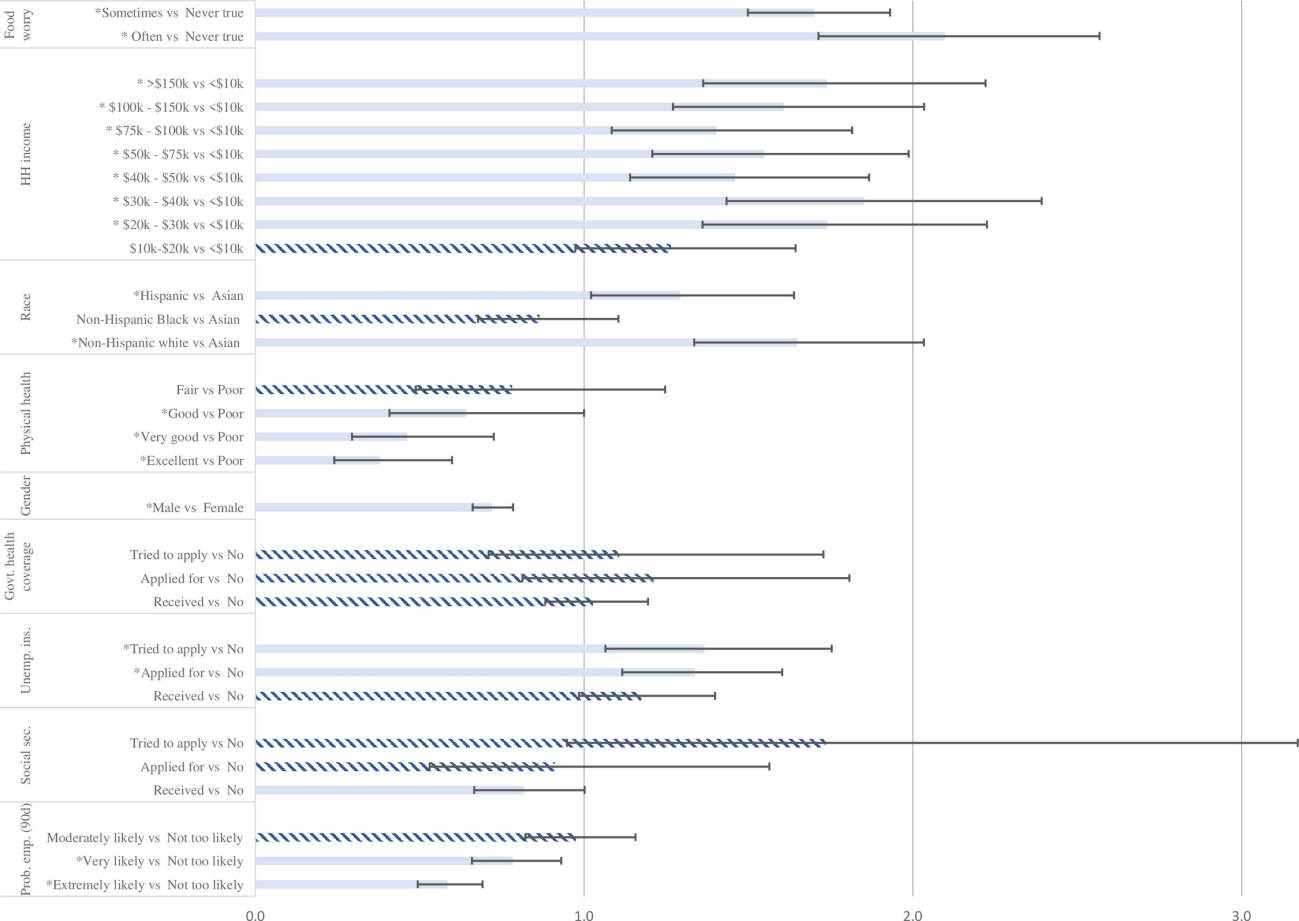
Odds-ratios of selected effect on moderate mental distress. The figure shows the odd ratios (and 95% confidence interval bars) for several economic-related variables obtained from a cross-sectional logistic regression of mental distress dummy. Coefficients with * and solid columns are significantly different from one at the 95% confidence level. Coefficients in striped columns are not statistically different from 1. Full regression results are presented in Table 1.

Next, we estimate a multivariate logistic regression of the moderate mental distress indicator and the economic and finance uncertainty variables. The full regression results and all relevant survey questions are presented in Table 1. A subset of the relevant estimated odd-ratios (OR) and 95% confidence intervals are presented in Fig 2. Odds ratios above (below) 1 imply a higher (lower) likelihood of mental distress. The results remain qualitatively unchanged when using the continuous composite measure T5, presented in S6 Table in S1 File.

**Table 1:** Logit regression odd ratios.

Effect	OR	CI 95%	
**Household structure**	** **	** **	** **
Alone vs 3 or more kids	1.702	1.439	2.013
plus 1 other adult only vs 3 or more kids	1.222	1.035	1.443
1 or 2 kids vs 3 or more kids	1.096	0.932	1.289
**Household income**			
$10,000 to under $20,000 vs Under $10,000	1.264	0.973	1.643
$20,000 to under $30,000 vs Under $10,000	1.739	1.360	2.225
$30,000 to under $40,000 vs Under $10,000	1.851	1.433	2.391
$40,000 to under $50,000 vs Under $10,000	1.459	1.140	1.867
$50,000 to under $75,000 vs Under $10,000	1.548	1.207	1.987
$75,000 to under $100,000 vs Under $10,000	1.403	1.084	1.815
$100,000 to under $150,000 vs Under $10,000	1.607	1.270	2.034
$150,000 or more vs Under $10,000	1.739	1.362	2.221
**Gender**			
Male vs Female	0.720	0.661	0.784
**Would you say your health in general is excellent, very good, good, fair, or poor?**			
Excellent vs Poor	0.378	0.240	0.598
Very good vs Poor	0.462	0.294	0.725
Good vs Poor	0.642	0.408	1.000
Fair vs Poor	0.780	0.488	1.246
**Race**			
Non-Hispanic white vs Non-Hispanic Asian	1.647	1.335	2.033
Non-Hispanic Black vs Non-Hispanic Asian	0.864	0.677	1.104
Hispanic vs Non-Hispanic Asian	1.293	1.021	1.638
**Age**			
18–24 vs 75+	5.166	3.459	7.713
25–34 vs 75+	3.358	2.310	4.880
35–44 vs 75+	3.020	2.076	4.393
45–54 vs 75+	2.048	1.408	2.978
55–64 vs 75+	1.441	0.998	2.082
65–74 vs 75+	1.055	0.735	1.512
**[A mental health condition] Has a doctor or other health care provider ever told you, you have any of the following?**			
Yes vs No	3.699	3.311	4.134
**Think about 3 months from now, how likely do you think it is that you will be employed at that time?**			
Extremely likely vs Not too likely	0.584	0.494	0.691
Very likely vs Not too likely	0.783	0.659	0.930
Moderately likely vs Not too likely	0.974	0.821	1.156
**[Social Security] In the past 7 days, have you either received, applied for, or tried to apply for any of the following forms of income or assistance, or not?**			
Received vs Did not receive nor apply for any	0.816	0.666	1.001
Applied for vs Did not receive nor apply for any	0.910	0.530	1.563
Tried to apply for vs Did not receive nor apply for any	1.734	0.948	3.170
**[Unemployment insurance] In the past 7 days, have you either received, applied for, or tried to apply for any of the following forms of income or assistance, or not?**			
Received vs Did not receive nor apply for any	1.173	0.984	1.398
Applied for vs Did not receive nor apply for any	1.337	1.116	1.602
Tried to apply for vs Did not receive nor apply for any	1.366	1.065	1.753
**In the past month, how often did you communicate with friends and family by phone, text, email, app, or using the Internet?**			
Basically every day vs Not at all	1.526	0.710	3.276
A few times a week vs Not at all	1.280	0.595	2.757
A few times a month vs Not at all	1.030	0.470	2.258
Once a month vs Not at all	1.138	0.474	2.728
**During a typical month prior to March 1, 2020, when COVID-19 began spreading in the United States, how often did you communicate with friends and family by phone, text, email, app, or using the Internet?**			
Basically every day vs Not at all	0.877	0.380	2.022
A few times a week vs Not at all	0.950	0.412	2.191
A few times a month vs Not at all	1.102	0.475	2.557
Once a month vs Not at all	1.253	0.511	3.072
**[We worried our food would run out before we got money to buy more] Please indicate whether the following statements were often true, sometimes true, or never true for you or your household over the past 30 days.**			
Often true vs Never true	2.097	1.713	2.567
Sometimes true vs Never true	1.700	1.498	1.930
**[Any kind of government health insurance or health coverage plan including Medicaid, Medical Assistance or Medicare] In the past 7 days, have you either received, applied for, or tried to apply for any of the following forms of income or assistance, or no**			
Received vs Did not receive nor apply for any	1.026	0.882	1.194
Applied for vs Did not receive nor apply for any	1.211	0.812	1.807
Tried to apply for vs Did not receive nor apply for any	1.107	0.709	1.728
**In the past 7 days, did you do any work for pay at a job or business?**			
Yes, I worked for someone else for wages, salary, piece rate, commission, tips, or payments ’in kind,’ for example, food or lodging received as payment for work performed vs No, I did not work for pay last week.	1.165	1.034	1.313
Yes, I worked as self-employed in my own business, professional practice, or farm vs No, I did not work for pay last week.	1.105	0.938	1.302
June fixed effect vs April	0.892	0.804	0.990
May fixed effect vs April	0.905	0.821	0.997

The table presents a cross-sectional logistic regression of the moderate mental distress indicator variables on financial uncertainty, economic uncertainty, and other control variables.

It is clear from the graph that individuals with a high likelihood of having a job in the next 90 days were much less likely to report that they feel moderate distress (50% less likely than those not likely to have a job, OR = 0.58 95% CI 0.49–0.69). In addition, individuals with greater uncertainty (tried to apply but did not manage) about social security (OR = 1.73 95% CI 0.95–3.17) and unemployment insurance (OR = 1.37 95% CI 1.07–1.75) were more likely to feel mentally distressed as compared with those who did not apply and with those who received support. Finally, those who had major worries (often true) about being able to afford food: [We worried our food would run out before we got money to buy more], were more likely (OR = 2.10 95% CI 1.73–2.57) to feel mentally distressed than those who did not have such concerns.

We also found that demographic characteristics were strongly related to mental health. Men were 20% less likely than women to feel moderate distress (OR = 0.72 95% CI 0.66–0.78), and non-Hispanic whites (OR = 1.65 95% CI 1.34–2.03) and Hispanics (OR = 1.29 95% CI 1.02–1.64) were 50% more likely than ethnic Asians to report moderate mental distress. Finally, those in excellent (OR = 0.38 95% CI 0.24–0.60), very good (OR = 0.46 95% CI 0.29–0.73), or good (OR = 0.64 95% CI 0.41–1.00) physical health were much less likely to feel moderate distress.

## Discussion

At a time of heightened public health crisis as well as economic uncertainty, it is essential to safeguard the mental wellness of the population with effective, sensitive, and timely public policies for potentially silent sources of universal psychological distress, which will likely impair the occupational and social functioning of the population for an extended period. Our findings support and complement previous results that economic downturns are related to deteriorating mental health [13, 25, 26].

The COVID-19 related public healthcare and economic interventions have been swift and massive. However, the interventions have been broad based and unconditional. Many governments around the world have responded to the impact of COVID-19 by implementing mental health interventions that address common mental health disorders, but there are acute shortages of such services in most countries (WHO, 2020). Most of these initiatives have involved investing in increased resources for traditional mental health services, as well as boosting capacity for telehealth mental health services that are required in the context of social isolation and quarantining, which unfortunately have had low uptake in most countries [11].

However, the current data highlight that, at the population level, one of the major initiatives that governments could be planning to achieve better mental health is investing in programs that address economic uncertainty. Many countries are already investing in facilitating economic recovery following the hugely disruptive impact of the pandemic, with limited success as the global economy is set to shrink by 4.4%. However, this study suggests that focusing on targeted economic programs for individuals who are most at risk of mental health problems may be particularly practical, effective, and helpful.

These findings also point to certain groups of the community who may be particularly vulnerable to mental health problems during COVID-19. Females were disproportionately affected, which accords with substantial evidence that females are more at risk of developing common mental disorders, such as anxiety and depression [27, 28]. There is also evidence emerging from studies during COVID-19 that females may be experiencing greater financial stress, which may compound their greater vulnerability to developing mental health problems during and after the pandemic. Similarly, there is evidence of race being related to mental health problems. This finding underscores that individuals from specific racial backgrounds may be more at risk of developing mental health problems through greater exposure to protracted downturns in the economy. Consequently, it is important to condition (control) for demographic information in calculating odds ratios related to mental disorders.

### Implications

The survey evidence presented here shows that economic and financial uncertainty, i.e., not knowing the extent of help available during a pandemic, is highly correlated with higher levels of mental distress. This main finding strongly suggests that economic policies (e.g., job keeping, job search support, stimulus spending) which remove economic and financial uncertainty and provide economic security, may also have externalities and constitute a major mental health intervention. The findings point to targeted economic policies complementing mental health interventions to address moderate forms of mental distress.

## Strengths and limitations

The dataset analyzed in this study is different from most mental health surveys that have been used during COVID-19, because it is based on a large representative sample that permits conclusions about the relation among COVID-19, economic effects, and mental health effects. Furthermore, the current economic shock is exogenous to mental health, such that an individual’s mental health has little or no contemporaneous or sequential impact on the immediate state of their economic and job prospects. In this setting, the direction of causality is likely to run from factors that are directly associated with economic conditions to mental health outcomes conditional on other demographic information, which may allow for cleaner identification of the interconnected relation between economic outcomes and mental health. However, results should be taken with caution due to several aspects.

First, a diagnostic evaluation for anxiety disorder or depressive disorder was not conducted directly. However, clinically validated screening instruments were used to assess five symptoms. Second, while the study controls for many factors that affect mental disorders, there may be factors related to COVID-19 that have not been included in the analysis, which may lead to omitted variable bias. Third, the nature of the crisis and of the analysis is suggestive of a causal relation between economic uncertainty and CMDs, but it is not conclusive. Indeed, the current economic conditions the respondents find themselves in may not be directly related to their ability or their mental health, but the overall state of the economy spurred by the health crisis. However, we cannot rule out the possibility of other potential confounders affecting both individual mental health and their economic uncertainty that have not been accounted for in the analysis. Fourth, while the data analyzed is representative of the U.S. population, the identified effects may vary across countries. There may be heterogeneity in the magnitude of the effect for countries at different levels of economic development and that were impacted differently by the COVID-19 pandemic.

## Conclusion

The depth of the current global economic crisis is almost unparalleled in modern history. Our study is important in deriving effective, sensitive, and timely public policies for potentially silent sources of universal psychological distress, which will likely impair the occupational and social functioning of the population for an extended period. Country-wide and community-level intervention and prevention efforts should include strengthening economic support to reduce financial and economic uncertainty and strain. For example, the data indicate that specific attention needs to be focused on reducing economic uncertainty deriving from the provision of social security, health and unemployment insurance. Speedy resolutions of applications for health and unemployment insurance would reduce mental distress. Furthermore, there is the need for more economic policies which support employment (e.g., job keeping, job search support, stimulus spending) and reduce economic uncertainty and mental distress.

## Supporting information

S1 File (DOCX)
