# Supplementary material for: Common Mental Disorders and Economic Uncertainty: Evidence from the COVID-19 Pandemic in the U.S
Source: PLoS One. 2021 Dec 2;16(12):e0260726. doi: 10.1371/journal.pone.0260726 (PMC8638947; doi:10.1371/journal.pone.0260726)
Supplement: S1 File — (DOCX) [file pone.0260726.s001.docx]

Supplementary Materials for

COMMON MENTAL DISORDERS AND ECONOMIC UNCERTAINTY: EVIDENCE FROM THE COVID-19 PANDEMIC IN THE U.S.

Wing Wah Tham, Elvira Sojli*, Richard Bryant, Michael McAleer

*Corresponding Author: E-mail: [e.sojli@unsw.edu.au](mailto:e.sojli@unsw.edu.au)

**This PDF file includes:**

S1 Sample characteristics

Figures S1 to S3

Tables S1 to S6

**S1. Sample Characteristics**

Supplementary Table S2 provides a summary of the survey participants characteristics. 48% of the sample comprises males, while 64% of the sample comprises white non-Hispanics. The largest age-group category among respondents is the above 60 years old (32%), followed by the 30-44 years old (30%). 64% of the sample live in a household with income below $75,000, which is consistent with the median household income of $63,000. One-third of the respondents (32%) live alone, 26% percent live in a shared household without children, and 26% have children in their household. Most of the survey participants report to be in good or very good health (72%), 12% report to be in excellent physical health, 13% report fair health, and 3% report poor health.

Supplementary Table S3 present the prevalence rates of moderate mental distress across the categories, and the differences between groups. The mean prevalence rate for the sample is 39.62%. 43% of females exhibit moderate mental distress in comparison to 36% of males. The difference across the groups is 7% and is statistically different from zero. Non-white Hispanics have a moderate mental distress rate of 43% in comparison with non-whites of 36%. The 7% difference is significant. As found in previous studies, mental distress decreases with age, where 18-29 years old exhibit a 52% rate of moderate mental distressed compared with 23% for the 60+ years old. The 29% difference is statistically significant. There is no statistical difference in mental distress across the very high (39%) and very low (37%) household income groups.

There is a significant difference of 5% and 6% in moderate mental distress between living alone and living with either other adults or with adults and children, respectively. Respondents in poor physical health have a 61% prevalence rate of moderate mental distress as compared with those in excellent health, who exhibit a 37% prevalence rate. Finally, respondents with a previously diagnosed mental illness have a 73% prevalence rate in moderate mental distress, which is consistent with the analysis in the previous section.

Supplementary Table S5 provides the summary information on various characteristics of interest, and the differences across those characteristics for three alternative cut-off points for the moderate mental distress variable. Overall, individuals who face threats and uncertainty related to unemployment, financial circumstances, social security, and health insurance, are systematically more mentally distressed. Individuals with poor physical health, low household income, and pre-existing mental illness are particularly susceptible to greater mental stress. Young workers and women are also more vulnerable.


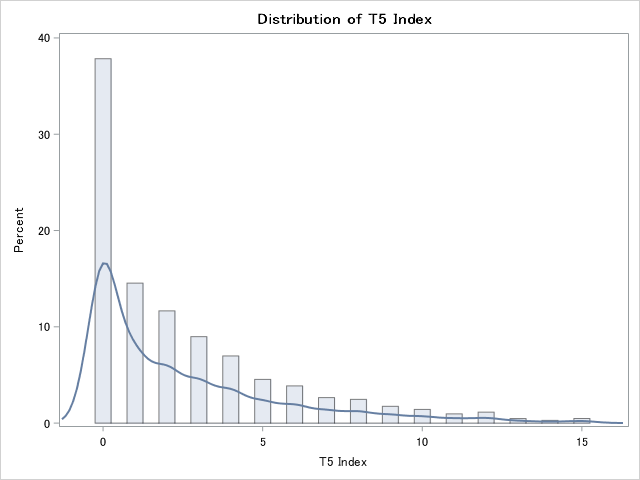


**S1 Fig. Composite measure T5 histogram.**

The figure presents the histogram of the composite measure T5. The sample comprises 11,480 respondents in three survey waves: 20-26 April 2020, 4-10 May 2020, 30 May-8 June 2020.


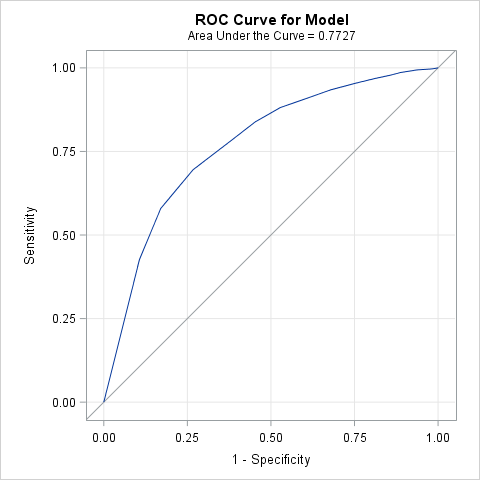


**S2 Fig. ROC curve for composite score and mental illness diagnosis.**

The receiver operating characteristic (ROC) curve is a plot of the true positives (sensitivity) versus the false positives (1 – specificity) for a binary clinical outcome classifier system as its screening test discrimination threshold is varied. The clinical outcome is whether the respondent reported that a doctor or other health care providers ever told them that they have a mental health condition (mental health diagnosis); the screening test is the T5 scale. The sample comprises 11,480 respondents in three survey waves: 20-26 April 2020, 4-10 May 2020, 30 May-8 June 2020.

Panel **a**. Employment

Panel **b**. Financial and economic uncertainty

**S3 Fig. Alternative moderate mental distress threshold (score ≥ 5).**

**S1 Table. Summary statistics of mental health variables.**

*Panel A. Variable Characteristics*

| In the past 7 days, how often have you? | Mean | Std. Dev. |
| --- | --- | --- |
| Felt nervous, anxious, or on edge | 0.64 | 0.91 |
| Felt depressed | 0.65 | 0.92 |
| Felt lonely | 0.65 | 0.92 |
| Felt hopeless about the future | 0.64 | 0.91 |
| Had physical reactions such as sweating, trouble breathing, nausea or a pounding heart when thinking about your experience with the coronavirus pandemic | 0.15 | 0.50 |
| Mental health composite score, T5 | 2.73 | 3.24 |

*Panel B. Correlations*

|  | Composite | Nervous | Depressed | Lonely | Hopeless |
| --- | --- | --- | --- | --- | --- |
| Nervous | 0.80** |  |  |  |  |
| Depressed | 0.81** | 0.56** |  |  |  |
| Lonely | 0.81** | 0.55** | 0.56** |  |  |
| Hopeless | 0.81** | 0.54** | 0.55** | 0.57** |  |
| Physical reaction | 0.57** | 0.36** | 0.36** | 0.36** | 0.38** |

*Panel C. Cronbach’s Coefficient Alpha*

| Standardized | 0.82 | Raw | 0.82 |
| --- | --- | --- | --- |

*Panel D. Cronbach’s Coefficient Alpha with Deleted Variable*

| Deleted Variable | Raw | Standardized |
| --- | --- | --- |
| Nervous | 0.77 | 0.78 |
| Depressed | 0.77 | 0.77 |
| Lonely | 0.77 | 0.77 |
| Hopeless | 0.77 | 0.77 |
| Physical reaction | 0.83 | 0.83 |

** denote statistically difference from zero with p-value <0.01.

**S2 Table. Sample characteristics.**

|  |  | % | S.E. (in %) |
| --- | --- | --- | --- |
| Gender | Male | 48 | 0.75 |
|  | Female | 52 | 0.75 |
| Race | White non-Hispanic | 64 | 0.73 |
|  | Non-white | 36 | 0.73 |
| Age | 18-29 | 15 | 0.54 |
|  | 30-44 | 30 | 0.69 |
|  | 45-59 | 24 | 0.64 |
|  | 60+ | 32 | 0.70 |
| Household Income | <$10k | 5 | 0.34 |
|  | $10k - $20 | 9 | 0.43 |
|  | $20k - $30k | 12 | 0.50 |
|  | $30k - $40k | 10 | 0.44 |
|  | $40k - $50k | 9 | 0.42 |
|  | $50k - $75k | 19 | 0.60 |
|  | $75k - $100k | 14 | 0.52 |
|  | $100k - $150k | 14 | 0.52 |
|  | >$150k | 7 | 0.40 |
| Household Composition | Alone | 32 | 0.70 |
|  | +1 other adult only | 26 | 0.66 |
|  | 1 or 2 kids | 17 | 0.57 |
|  | 3 or more kids | 9 | 0.43 |
|  | Other | 16 | 0.55 |
| Physical Health | Excellent | 12 | 0.49 |
|  | Very good | 39 | 0.73 |
|  | Good | 33 | 0.71 |
|  | Fair | 13 | 0.51 |
|  | Poor | 3 | 0.23 |

The table presents the sample composition in percent of the sample (%) and the standard error for each category (S.E. in %) for gender, race, age group, household income, household composition, and physical health.

**S3 Table. Moderate mental distress prevalence rates across groups.**

| Variables | Group | Prevalence rate | Diff. |
| --- | --- | --- | --- |
|  | Sample | 40% |  |
| Gender | Male | 36% | -7%** |
|  | Female | 43% |  |
| Race | White non-Hispanic | 43% | 7%** |
|  | Non-white | 36% |  |
| Age | 18-29 | 52% | 29%** |
|  | 60+ | 23% |  |
| Household Income | < $10k | 37% | -2% |
|  | >$150k | 39% |  |
| Household Composition | Alone | 44% | 5%* |
|  | + other adults and kids | 39% |  |
|  | Alone | 44% | 6%* |
|  | + 1 other adult only | 37% |  |
| Physical Health | Poor | 61% | 25%** |
|  | Excellent | 37% |  |
| Existing mental illness | Yes | 73% | 40%** |
|  | No | 33% |  |

The table presents the prevalence of moderate mental distress (*Prevalence rate*) across different demographic and social characteristics. *Diff*. is the difference between the two groups.

**S4 Table. Moderate mental distress and change in working hours.**

| Work Hours Change /Week | | | |
| --- | --- | --- | --- |
| Mean | -3.5 hours |  |  |
| St. Dev. | 11 hours |  |  |
| Mental Distress Elasticity (/hour) | | | |
| 0.14% | T5 composite score ≥ 3 | |  |
| 0.13% | T5 composite score ≥ 4 | |  |
| 0.11% | T5 composite score ≥ 5 | |  |
| 0.013 | T5 composite score | |  |
| Effect of 1 St. dev. /week | | | |
| 1.54% | T5 composite score ≥ 3 | |  |
| 1.43% | T5 composite score ≥ 4 | |  |
| 1.21% | T5 composite score ≥ 5 | |  |
| 0.143 | T5 composite score | |  |

**S5 Table. Differences in moderate mental distress across individual characteristics.**

*Panel A. Demographics*

|  | Gender | | | Race | | | Age | | | | | Household Composition | | | | | |
| --- | --- | --- | --- | --- | --- | --- | --- | --- | --- | --- | --- | --- | --- | --- | --- | --- | --- |
| Threshold | Male | Female | Diff. | White non-Hispanic | Non-white | Diff. | 18-29 | 30-44 | 45-59 | 60+ | Diff. 18-29 vs. 60+ | Alone | +1 adult only | Diff. | Alone | + adult  + kids | Diff. |
| ≥ 3 | 36 | 43 | -7** | 43 | 36 | 7** | 52 | 43 | 33 | 23 | 29** | 44 | 37 | 6* | 44 | 39 | 5 |
| ≥ 4 | 29 | 34 | -5* | 34 | 28 | 6* | 44 | 33 | 26 | 17 | 27** | 34 | 29 | 5* | 34 | 31 | 3 |
| ≥ 5 | 20 | 27 | -6** | 25 | 22 | 3 | 36 | 23 | 20 | 12 | 24** | 26 | 21 | 5* | 26 | 24 | 3 |

*Panel B. Individual characteristics*

|  | Household Income | | | Physical Health | | | Existing Mental Illness | | | Communication with Others | | |
| --- | --- | --- | --- | --- | --- | --- | --- | --- | --- | --- | --- | --- |
| Threshold | <$10k | >$150k | Diff. | Poor | Excellent | Diff. | Yes | No | Diff. | Few times/week | Max. few times/month | Diff. |
| ≥ 3 | 37 | 39 | -2 | 61 | 37 | 25* | 73 | 33 | 40** | 37 | 34 | 3 |
| ≥ 4 | 25 | 32 | -7 | 59 | 28 | 31** | 64 | 25 | 39** | 28 | 31 | -3 |
| ≥ 5 | 18 | 26 | -8 | 48 | 19 | 29** | 55 | 17 | 37** | 20 | 20 | 0 |

*Panel C. Finance and economics*

|  | Financially | | | Unemployed | | | Likely Future Employment | | | | | | Social Security Application | | | Medical Ins. & Assistance App. | | | Worry about Food | | |
| --- | --- | --- | --- | --- | --- | --- | --- | --- | --- | --- | --- | --- | --- | --- | --- | --- | --- | --- | --- | --- | --- |
|  | Constrained | | |  |  |  | (30 days) | | | (90 days) | | |  |  |  |  |  |  |  |  |  |
| Threshold | Yes | No | Diff. | Yes | No | Diff. | High | Low | Diff. | High | Low | Diff. | Applied/  received/  did not apply | Tried to apply | Diff. | Applied/  received/  did not apply | Tried to apply | Diff. | Never worry | Often or sometimes worry | Diff. |
| ≥ 3 | 49 | 38 | 12** | 44 | 37 | 7** | 37 | 49 | -12** | 38 | 50 | -12** | 39 | 85 | -46** | 39 | 72 | -33** | 35 | 49 | -14** |
| ≥ 4 | 41 | 29 | 11** | 36 | 29 | 7** | 29 | 41 | -12** | 30 | 46 | -16** | 31 | 79 | -48** | 31 | 67 | -36** | 27 | 41 | -14** |
| ≥ 5 | 32 | 22 | 10** | 28 | 21 | 7** | 21 | 33 | -12** | 22 | 36 | -14** | 23 | 66 | -43** | 23 | 57 | -34** | 19 | 33 | -14** |

All data is in %. * and ** denote differences (*Diff.*) are statistically different from zero with p-value <0.05 and <0.01 respectively.

**S6 Table. Logit regression odd ratios for T5 threshold score ≥ 5.**

| **Effect** | **OR** | **CI 95%** | |
| --- | --- | --- | --- |
| **Household structure** |  |  |  |
| Alone vs 3 or more kids | 1.596 | 1.393 | 1.829 |
| plus 1 other adult only vs 3 or more kids | 1.166 | 1.019 | 1.334 |
| 1 or 2 kids vs 3 or more kids | 1.074 | 0.942 | 1.225 |
| **Household income** |  |  |  |
| $10,000 to under $20,000 vs Under $10,000 | 1.275 | 1.035 | 1.571 |
| $20,000 to under $30,000 vs Under $10,000 | 1.781 | 1.462 | 2.17 |
| $30,000 to under $40,000 vs Under $10,000 | 1.909 | 1.555 | 2.344 |
| $40,000 to under $50,000 vs Under $10,000 | 1.379 | 1.131 | 1.681 |
| $50,000 to under $75,000 vs Under $10,000 | 1.580 | 1.294 | 1.929 |
| $75,000 to under $100,000 vs Under $10,000 | 1.351 | 1.098 | 1.662 |
| $100,000 to under $150,000 vs Under $10,000 | 1.622 | 1.343 | 1.958 |
| $150,000 or more vs Under $10,000 | 1.731 | 1.423 | 2.105 |
| **Gender** |  |  |  |
| Male vs Female | 0.707 | 0.660 | 0.758 |
| **Would you say your health in general is excellent, very good, good, fair, or poor?** |  |  |  |
| Excellent vs Poor | 0.304 | 0.215 | 0.432 |
| Very good vs Poor | 0.376 | 0.266 | 0.53 |
| Good vs Poor | 0.494 | 0.349 | 0.697 |
| Fair vs Poor | 0.716 | 0.500 | 1.024 |
| **Race** |  |  |  |
| Non-Hispanic white vs Non-Hispanic Asian | 1.683 | 1.419 | 1.997 |
| Non-Hispanic Black vs Non-Hispanic Asian | 0.883 | 0.725 | 1.076 |
| Hispanic vs Non-Hispanic Asian | 1.241 | 1.024 | 1.504 |
| **Age** |  |  |  |
| 18-24 vs 75+ | 5.997 | 4.403 | 8.167 |
| 25-34 vs 75+ | 3.812 | 2.864 | 5.074 |
| 35-44 vs 75+ | 3.220 | 2.417 | 4.29 |
| 45-54 vs 75+ | 2.259 | 1.697 | 3.008 |
| 55-64 vs 75+ | 1.597 | 1.207 | 2.112 |
| 65-74 vs 75+ | 1.209 | 0.922 | 1.586 |
| **[A mental health condition] Has a doctor or other health care provider ever told you, you have any of the following?** |  |  |  |
| Yes vs No | 3.995 | 3.646 | 4.377 |
| **Think about 3 months from now, how likely do you think it is that you will be employed at that time?** |  |  |  |
| Extremely likely vs Not too likely | 0.548 | 0.479 | 0.627 |
| Very likely vs Not too likely | 0.781 | 0.680 | 0.897 |
| Moderately likely vs Not too likely | 0.934 | 0.815 | 1.072 |
| **[Social Security] In the past 7 days, have you either received, applied for, or tried to apply for any of the following forms of income or assistance, or not?** |  |  |  |
| Received vs Did not receive nor apply for any | 0.853 | 0.728 | 1.000 |
| Applied for vs Did not receive nor apply for any | 0.776 | 0.511 | 1.180 |
| Tried to apply for vs Did not receive nor apply for any | 2.189 | 1.416 | 3.385 |
| **[Unemployment insurance] In the past 7 days, have you either received, applied for, or tried to apply for any of the following forms of income or assistance, or not?** |  |  |  |
| Received vs Did not receive nor apply for any | 1.207 | 1.048 | 1.390 |
| Applied for vs Did not receive nor apply for any | 1.348 | 1.165 | 1.559 |
| Tried to apply for vs Did not receive nor apply for any | 1.321 | 1.080 | 1.616 |
| **In the past month, how often did you communicate with friends and family by phone, text, email, app, or using the Internet?** |  |  |  |
| Basically every day vs Not at all | 2.119 | 1.155 | 3.886 |
| A few times a week vs Not at all | 1.730 | 0.941 | 3.181 |
| A few times a month vs Not at all | 1.348 | 0.723 | 2.511 |
| Once a month vs Not at all | 1.318 | 0.661 | 2.629 |
| **During a typical month prior to March 1, 2020, when COVID-19 began spreading in the United States, how often did you communicate with friends and family by phone, text, email, app, or using the Internet?** |  |  |  |
| Basically every day vs Not at all | 0.668 | 0.344 | 1.297 |
| A few times a week vs Not at all | 0.758 | 0.390 | 1.471 |
| A few times a month vs Not at all | 0.917 | 0.470 | 1.787 |
| Once a month vs Not at all | 0.956 | 0.470 | 1.945 |
| **[We worried our food would run out before we got money to buy more] Please indicate whether the following statements were often true, sometimes true, or never true for you or your household over the past 30 days.** |  |  |  |
| Often true vs Never true | 2.652 | 2.256 | 3.118 |
| Sometimes true vs Never true | 1.669 | 1.505 | 1.849 |
| **[Any kind of government health insurance or health coverage plan including Medicaid, Medical Assistance or Medicare] In the past 7 days, have you either received, applied for, or tried to apply for any of the following forms of income or assistance, or no** |  |  |  |
| Received vs Did not receive nor apply for any | 1.041 | 0.924 | 1.172 |
| Applied for vs Did not receive nor apply for any | 1.280 | 0.930 | 1.763 |
| Tried to apply for vs Did not receive nor apply for any | 1.315 | 0.926 | 1.867 |
| **In the past 7 days, did you do any work for pay at a job or business?** |  |  |  |
| Yes, I worked for someone else for wages, salary, piece rate, commission, tips, or payments 'in kind,' for example, food or lodging received as payment for work performed vs No, I did not work for pay last week. | 1.202 | 1.092 | 1.324 |
| Yes, I worked as self-employed in my own business, professional practice, or farm vs No, I did not work for pay last week. | 1.156 | 1.015 | 1.318 |
| June fixed effect vs April | 0.920 | 0.846 | 1.001 |
| May fixed effect vs April | 0.897 | 0.83 | 0.971 |

The table presents a cross-sectional logistic regression of the moderate mental distress composite measure T5 on financial uncertainty, economic uncertainty, and other control variables.
